# Risk Factors for Serogroup C Meningococcal Disease during Outbreak among Men who Have Sex with Men, New York City, New York, USA

**DOI:** 10.3201/eid2108.141932

**Published:** 2015-08

**Authors:** Alison Ridpath, Sharon K. Greene, Byron F. Robinson, Don Weiss, the Meningococcal Investigation Team

**Affiliations:** New York City Department of Health and Mental Hygiene, Queens, New York, USA; (A. Ridpath, S.K. Greene, D. Weiss);; Centers for Disease Control and Prevention, Atlanta, Georgia, USA (A. Ridpath, B.F. Robinson)

**Keywords:** Neisseria meningitidis, bacteria, invasive meningococcal disease, serogroup C, disease outbreaks, case–control study, risk factors, homosexuality, men who have sex with men, New York City, New York

## Abstract

Risk factors for illness during a serogroup C meningococcal disease outbreak among men who have sex with men in New York City, New York, USA, in 2012–2013 included methamphetamine and cocaine use and sexually transmitted infections. Outbreak investigations should consider routinely capturing information regarding drug use and sex-related risk factors.

In the United States, meningococcal disease, a nationally reportable bacterial disease caused by *Neisseria meningitidis*, has a case-fatality rate of 10%–15% (*1*). Clusters of serogroup C meningococcal disease have been reported among men who have sex with men (MSM) in Chicago, Illinois, USA; Toronto, Ontario, Canada; and Europe (*2**–**4*). However, case–control studies to evaluate unique risk factors among this population are lacking.

During August 2010–February 2013, New York City (NYC), New York, had a protracted outbreak of serogroup C meningococcal disease among MSM, which is described elsewhere in this issue (*5*). To more fully understand risk factors associated with the outbreak, the NYC Department of Health and Mental Hygiene (DOHMH) conducted a case–control study.

## The Study

An outbreak case was defined as an illness clinically compatible with serogroup C meningococcal disease meeting the 2010 Council of State and Territorial Epidemiologists case definition for a confirmed or probable case (*6*) with onset during January 2012–February 2013 in NYC male residents self-identifying as gay or bisexual or reporting sexual contact with another man during the previous year. Meningococcal disease case investigations include interviews with the patient (when possible), health care providers, family members, and close contacts and review of the patient’s medical records. Controls were selected from NYC male residents given a diagnosis of infection with *Giardia lamblia* (giardiasis) or *Entamoeba histolytica* (amebiasis) during January 2012–February 2013, who had not been routinely interviewed by DOHMH. Three controls were matched to each case-patient for age at disease diagnosis (± 5 years) and diagnosis date (± 1 month). Controls were ineligible if they were not NYC residents, were non-English speaking, had invasive meningococcal disease during the study, or were non-MSM.

During March–April 2013, case-patients and controls were interviewed by telephone by using a 20-min questionnaire after informed consent was obtained. The questionnaire elicited information regarding demographic features and lifestyle (e.g., alcohol and drug use, smoking, bar and party attendance, and number and ways of meeting sexual contacts). Case-patients and controls were asked about exposures during the 30 days before illness onset. Questionnaires for deceased or unreachable case-patients were completed on the basis of information obtained during initial investigation. If drug use or sexual contact could not be determined, values for these patients were considered unknown and associated data were excluded from analysis. HIV and sexually transmitted infections (STIs) (i.e., chlamydia, gonorrhea, and syphilis) for case-patients and controls were obtained from DOHMH registries on June 5, 2013.

All epidemiologic data were entered into a Microsoft Access 2010 database (Microsoft, Redmond, WA, USA). Statistical analyses were conducted by using SAS version 9.2 (SAS Institute, Cary, NS, USA). Matched odds ratios and 95% CIs were calculated by using conditional logistic regression and the mid-p exact method (*7*). Median unbiased estimates of the odds ratio were reported when warranted. HIV infection, previously reported to be a risk factor for meningococcal disease (*8*), was controlled for in the analysis.

Seventeen outbreak cases occurred during 2012 (n = 13) and 2013 (n = 4). Among 11 surviving serogroup C meningococcal disease case-patients, 10 were re-interviewed and 1 could not be reached. Of 90 possible controls, 51 eligible controls completed interviews. Of the remaining 39 possible controls, 6 were unreachable (after >5 call attempts, including evenings or weekends); 11 refused; 21 were ineligible (12 were non-MSM, 6 were non-English speakers, 2 were non-NYC residents, and 1 had a false-positive report); and 1 did not complete the interview. Unmatched characteristics of case-patients and controls are shown in Table 1. Most case-patients were Brooklyn residents, black, and HIV infected, and most controls were Manhattan residents, white, and non–HIV infected. Case-patients appeared to be of lower socioeconomic status, as indicated by income, education, and health insurance status.

**Table 1:** Characteristics of case-patients with outbreak-related serogroup C meningococcal disease and controls with amebiasis or giardiasis, New York City, New York, USA, 2012–2013*

Characteristic	Case-patients, n = 17	Controls, n = 51
Median age, y (range)	32 (21–59)	30 (22–59)
Borough (1 undomiciled case-patient excluded)		
Brooklyn	9/16 (56.3)	11/51 (21.6)
Manhattan	5/16 (31.3)	30/51 (58.8)
Queens or Bronx	2/16 (12.5)	10/51 (19.6)
Sexual orientation (self-identified)		
Gay	15/17 (88.2)	45/50 (90.0)
Bisexual	2/17 (11.8)	5/50 (10.0)
Race		
Black	10/17 (58.8)	5/50 (10.0)
White	5/17 (29.4)	42/50 (84.0)
Other	2/17 (11.8)	3/50 (6.0)
Hispanic	3/17 (17.6)	9/51 (17.6)
Employed	12/17 (70.6)	41/51 (80.4)
Annual household income		
≤$29,999	9/13 (69.3)	15/46 (32.6)
$30,000–$59,999	3/13 (23.1)	8/46 (17.4)
≥$60,000	1/13 (7.7)	23/46 (50.0)
Health insurance		
Yes	9/14 (64.3)	48/51 (94.1)
No	5/14 (35.7)	3/51 (5.9)
Education		
High school, GED, or less	5/15 (33.3)	5/50 (10.0)
At least some college	10/15 (66.7)	45/50 (90.0)
HIV infected	10/17 (58.8)	13/51 (25.5)

*Values are no. responded/total (%) unless otherwise indicated. Denominators exclude unknown and refused answers. GED, general educational development.

Matched odds ratios (crude and adjusted) are shown in Table 2. After we adjusted for HIV infection, exposures that remained independently associated with serogroup C meningococcal disease were black race, methamphetamine or cocaine use during the month before illness onset, and STI during the year before diagnosis. During the month before illness onset, tobacco smoking, sharing drinks, having sex with >1 man, or meeting a sex partner online or at a bar or party, although more common among case-patients, were not major risk factors during this outbreak.

**Table 2:** Risk factors for meningococcal disease among case-patients with outbreak-related serogroup C meningococcal disease and controls with amebiasis or giardiasis, New York City, New York, USA, 2012–2013*

Variable	Case-patients, no. responded/total (%), n = 17	Controls, no. responded/total (%), n = 51	Crude matched odds ratio (95% CI)	Matched odds ratio adjusted for HIV infection (95% CI)
Black race	10/17 (58.8)	5/50 (10.0)	**12.0 (2.8–81.6)**	**8.0 (1.6–63.7)**
Household with >1 other person	9/16 (56.3)	10/51 (19.6)	**5.6 (1.5–27.0)**	3.7 (1.0–18.0)
Tobacco smoking	6/17 (35.3)	14/50 (28.0)	1.4 (0.4–4.5)	0.9 (0.2–3.3)
Shared a drink	5/11 (45.5)	14/39 (35.9)	1.1 (0.2–4.8)	1.2 (0.3–5.5)
Drug use in month before illness onset				
Marijuana	3/15 (20.0)	13/51 (25.5)	0.7 (0.1–2.9)	0.5 (0.1–2.4)
Methamphetamine	7/17 (41.2)†	0/50 (0)	**28.8 (5.6–∞)‡**	**16.6 (3.1–∞)‡**
Cocaine	4/14 (28.6)	0/51 (0)	**15.9 (2.7–∞)‡**	**11.2 (1.8–∞)‡**
Sexual risk				
Sex with >1 man during month before illness onset	8/13 (61.5)	18/51 (35.3)	2.6 (0.7–10.7)	2.8 (0.7–13.7)
Met a male sex partner during month before illness onset online or at bar or party versus other ways§	7/10 (70.0)	21/40 (52.5)	2.1 (0.5–11.6)	1.8 (0.4–10.6)
Chlamydia, gonorrhea, or syphilis diagnoses during the year before diagnosis date¶	9/17 (52.9)	6/51 (11.8)	**7.2 (2.0–33.8)**	**4.7 (1.2–23.5)**
HIV infected	10/17 (58.8)	13/51 (25.5)	**6.4 (1.5–45.1)**	NA

*Denominators exclude unknown and refused answers. Values in bold indicate mid-p exact p<0.05. NA, not applicable. †One patient answered don't know, but laboratory testing at hospital indicated methamphetamine use. ‡Median unbiased estimate of the odds ratio. §Other ways of meeting included work, school, through a friend, gym, sports group, sex party, or other. ¶Diagnosis date refers to the diagnosis date of serogroup C meningococcal disease for case-patients and diagnosis date of amebiasis or giardiasis for controls.

No case-patients had documentation of meningococcal vaccine before illness onset. We were unable to document vaccine receipt in controls, which limited our ability to examine the effect meningococcal vaccination may have had on the risk for meningococcal infection during this outbreak.

## Conclusions

Although rates of methamphetamine and cocaine use are not known specifically among MSM in NYC, during 2008–2009, a total of 3% of the general NYC population reported past-year cocaine use (*9*), which was much lower than the 29% reported use among case-patients. Through inhalation, these drugs can damage respiratory mucosa and increase susceptibility to meningococcal disease (*10*). In addition, drug use can be a social activity involving equipment sharing among users, thus increasing respiratory secretion exposure.

Chlamydia, gonorrhea, or syphilis during the year before diagnosis was also a risk factor during this outbreak, despite controls having been selected on the basis of having a disease (giardiasis or amebiasis) that can be transmitted sexually (*11**,**12*). Controls might have been overmatched for sexual behavior (i.e., number of sexual partners or high-risk sexual practices) to case-patients. Whether the source of exposure during MSM sexual contact is through oropharyngeal secretions or represents a novel mechanism (e.g., rectal carriage of *N. meningitidis*) is unknown. More research is needed to better understand the apparent overlap of STIs and meningococcal disease among MSM. This investigation highlights the usefulness for public health practice of collecting and recording information regarding sexual behavior among meningococcal patients, specifically cases among MSM.

Although black race is not a risk factor for meningococcal disease (*1*), black race appeared to be a risk factor during this outbreak and was also common (75%) among patients during a 2005–2006 outbreak in Brooklyn (*13*). Race might be a proxy for a social network or an unidentified cofactor of this outbreak and not a biological risk factor for meningococcal disease. Determining risk factors for infection to target prevention measures during an ongoing outbreak is challenging, and public health authorities often cannot wait for results of epidemiologic studies before acting. Previously identified risk factors (e.g., smoking, household crowding, or sharing drinks) (*14**,**15*) and behaviors used to target our vaccine recommendations (i.e., meeting men for sex at a bar, party, online, or through digital applications [*5*]) were not associated with this outbreak but were more common among case-patients and might still be associated with meningococcal disease. This study might have had insufficient power to detect such differences.

This case–control study, conducted in the context of a prolonged outbreak of serogroup C meningococcal disease among MSM in NYC, identified 2 key risk factors: having an STI during the year before diagnosis and having used methamphetamine or cocaine during the month before illness onset. We hypothesize these factors might be associated with membership in a common social network in which carriage of serogroup C meningococci is increased (*13*). Consideration can be given to collecting drug use and sexual identity data through routine meningococcal disease surveillance to recognize transmission clusters and to more fully understand if these risk factors are generalizable. Future studies of meningococcal disease outbreaks should consider including assessment of these risk factors.
